# Assessment of thoracic disc degeneration using dual-energy CT-based collagen maps

**DOI:** 10.1186/s41747-024-00500-x

**Published:** 2024-08-26

**Authors:** Simon Bernatz, Alexander Tom Hoppe, Leon David Gruenewald, Vitali Koch, Simon S. Martin, Lara Engelskirchen, Ivana Radic, Giuseppe Bucolo, Jennifer Gotta, Philipp Reschke, Renate M. Hammerstingl, Jan-Erik Scholtz, Tatjana Gruber-Rouh, Katrin Eichler, Thomas J. Vogl, Christian Booz, Ibrahim Yel, Scherwin Mahmoudi

**Affiliations:** 1 Department of Diagnostic and Interventional Radiology, University Hospital Frankfurt, Goethe University Frankfurt am Main, Frankfurt am Main, Germany; 2 Dr. Senckenberg Institute for Pathology, University Hospital Frankfurt, Goethe University Frankfurt am Main, Frankfurt am Main, Germany; 3 Institute for Diagnostic and Interventional Radiology, University Hospital Zurich, Zurich, Switzerland; 4 Department of Biomedical Sciences and Morphological and Functional Imaging, University of Messina, Messina, Italy

**Keywords:** Collagen, Intervertebral disc degeneration, Magnetic resonance imaging, Spine, Tomography (x-ray computed)

## Abstract

**Background:**

We evaluated the role of dual-energy computed tomography (DECT)-based collagen maps in assessing thoracic disc degeneration.

**Methods:**

We performed a retrospective analysis of patients who underwent DECT and magnetic resonance imaging (MRI) of the thoracic spine within a 2-week period from July 2019 to October 2022. Thoracic disc degeneration was classified by three blinded radiologists into three Pfirrmann categories: no/mild (grade 1–2), moderate (grade 3–4), and severe (grade 5). The DECT performance was determined using MRI as a reference standard. Interreader reliability was assessed using intraclass correlation coefficient (ICC). Five-point Likert scales were used to assess diagnostic confidence and image quality.

**Results:**

In total, 612 intervertebral discs across 51 patients aged 68 ± 16 years (mean ± standard deviation), 28 males and 23 females, were assessed. MRI revealed 135 no/mildly degenerated discs (22.1%), 470 moderately degenerated discs (76.8%), and 7 severely degenerated discs (1.1%). DECT collagen maps achieved an overall accuracy of 1,483/1,838 (80.8%) for thoracic disc degeneration. Overall recall (sensitivity) was 331/405 (81.7%) for detecting no/mild degeneration, 1,134/1,410 (80.4%) for moderate degeneration, and 18/21 (85.7%) for severe degeneration. Interrater agreement was good (ICC = 0.89). Assessment of DECT-based collagen maps demonstrated high diagnostic confidence (median 4; interquartile range 3–4) and good image quality (median 4; interquartile range 4–4).

**Conclusion:**

DECT showed an overall 81% accuracy for disc degeneration by visualizing differences in the collagen content of thoracic discs.

**Relevance statement:**

Utilizing DECT-based collagen maps to distinguish various stages of thoracic disc degeneration could be clinically relevant for early detection of disc-related conditions. This approach may be particularly beneficial when MRI is contraindicated.

**Key Points:**

- A total of 612 intervertebral discs across 51 patients were retrospectively assessed with DECT, using MRI as a reference standard.
- DECT-based collagen maps allowed thoracic disc degeneration assessment achieving an overall 81% accuracy with good interrater agreement (ICC = 0.89).
- DECT-based collagen maps could be a good alternative in the case of contraindications to MRI.

**Graphical Abstract:**

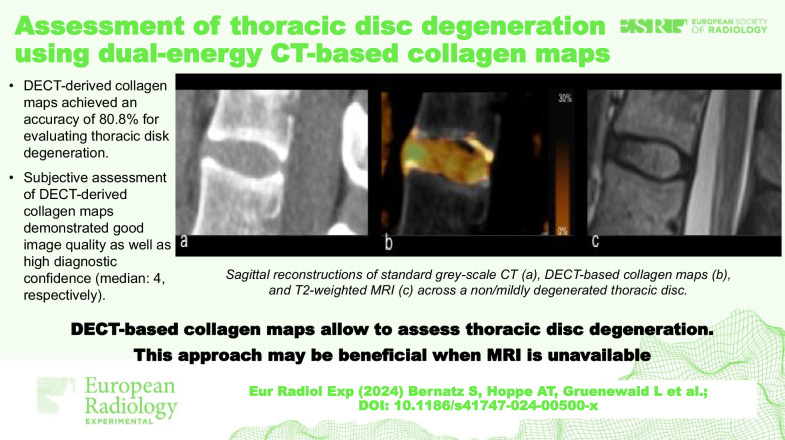

## Background

Intervertebral disc degeneration, characterized by loss of disc height and structural integrity, is presumably the first stage of spinal degenerative change and can present as pain [1–3]. Degenerative changes of the thoracic spine are highly prevalent, although much less common compared to the lumbar and cervical spine. Thoracic disc degeneration is considered mostly asymptomatic; however, the mechanics of the thoracic spine and the effects of respective degenerative changes are not yet fully understood, which can partly be attributed to the paucity of studies focusing on this spinal region [4–7].

Magnetic resonance imaging (MRI) is established as the preferred imaging modality for evaluating degenerative disc disease [8]. However, its limited availability, contraindications, and the necessity for patient cooperation call for the exploration of alternative imaging options. Dual-energy CT (DECT) has emerged as a relevant imaging technique in a broad spectrum of diseases by enabling material decomposition through analysis of tissue attenuation at different energy levels [9–13]. Musculoskeletal radiology offers various applications for DECT [14, 15]. It is well established in the imaging of gout and more recent studies have further investigated other DECT applications, such as discerning different crystal arthropathies and visualizing bone marrow edema [16–18]. In addition, DECT post-processing allows for collagen mapping of tissues through the quantification of hydroxyproline and hydroxylysine side chains contained in collagen molecules. Its feasibility in this regard has been studied, for instance, in knee ligaments and tendons of the hand and foot [19, 20]. Collagen is found in both sections of the intervertebral disc, with more collagen present in the outer annulus fibrosus compared to the inner nucleus pulposus, which mainly contains water and proteoglycans [21]. Systematic grading of intervertebral disc degeneration is commonly performed using the Pfirrmann classification and includes the assessment of discrimination between nucleus pulposus *versus* annulus fibrosus [22]. Two recent studies have explored the benefit of DECT collagen maps to evaluate degenerative changes in lumbar discs [23, 24]. One study has investigated its value for the assessment of disc injuries in the setting of thoracolumbar vertebrae fractures [25].

To date, no study has yet analyzed the diagnostic value of DECT collagen maps for evaluating disc degeneration in the thoracic spine. We hypothesized that DECT collagen maps can visualize differences in the collagen content of thoracic discs, hence providing information on the degree of degenerative change. The aim of this study was to analyze the diagnostic accuracy of DECT collagen maps for the evaluation of thoracic disc degeneration.

## Methods

The ethics review board of our institution approved this retrospective single-center study with a waiver for written informed consent.

### Study population

**Fig. 1:**
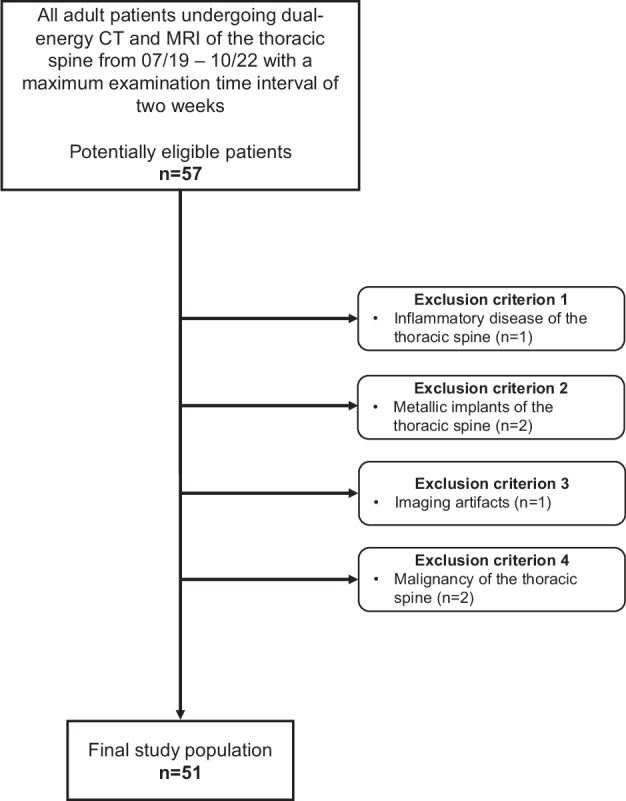
Flowchart of study inclusion

In this retrospective, single-center study, we conducted an examination of our database, focusing on patients who underwent dual-source DECT and MRI of the thoracic spine due to thoracic back pain following acute trauma from July 2019 to October 2022 with a maximum time span of 2 weeks between both examinations. We defined exclusion criteria as follows: (1) acute inflammatory process of the thoracic spine; (2) osteosynthesis of the thoracic spine; (3) studies with motion artifacts; and (4) documented malignant disease of the thoracic spine. Clinical data were extracted from medical records as part of routine clinical practice. The flowchart in Fig. 1 outlines the patient inclusion process, adhering to the Standards for Reporting Diagnostic Accuracy Studies (STARD) [26].

### DECT acquisition protocol and image reconstruction/post-processing

All DECT scans were conducted using a third-generation dual-source dual-energy CT scanner (Somatom Force, Siemens Healthineers, Munich, Germany) with standard parameters set as follows: tube A operated at 90 kVp with 220 mAs, and tube B operated at 150 kVp with 138 mAs, with an additional tin filter (Selective Photon Shield II, Siemens Healthineers). Scans were performed in craniocaudal direction with a rotation time of 500 ms and a collimation of 128 × 0.6 mm. The scanning protocol included automatic attenuation-based tube current modulation (CARE Dose 4D, Siemens Healthineers).

Each DECT scan generated two relevant image sets: one at 90 kVp and one at 150 kVp. Postprocessing was conducted using a commercially available syngo.via workstation (VB50, Siemens Healthineers). For collagen map reconstruction, the low-energy (90 kVp) and high-energy (150 kVp) image stacks were imported into the syngo.via workstation.

DECT collagen maps were reconstructed by a resident in radiology with 2 years of experience (J.G.) according to a vendor-specific application profile with the following parameters: collagen; display layout, fat map; width, 65: level, -30. The DECT-derived collagen maps were reconstructed with a slice thickness of 1 mm and an increment of 0.75 mm in both transversal and sagittal orientations. Subsequently, all image series were uploaded to the Picture Archiving and Communication System (PACS, GE Healthcare, Solingen, Germany) for subsequent analysis.

### Reference standard

In all participants, a reference standard noncontrast MRI was conducted on a 3-T system (Magnetom PrismaFit; Siemens Healthineers). The standard imaging sequences comprised T1-weighted spin-echo (repetition time 650 ms; echo time 10 ms; field of view 360 × 360 mm^2^; matrix size 288 × 384; section thickness 4 mm), T2-weighted fast spin-echo (repetition time 4,000 ms; echo time 89 ms; field of view 360 × 360 mm^2^; matrix size 358 × 448; section thickness 4 mm), and turbo inversion-recovery magnitude (inversion time 220 ms; repetition time 3,500 ms; echo time 39 ms; field of view 360 × 360 mm^2^; matrix size 388 × 384; section thickness,4 mm), all captured in the sagittal plane.

### DECT image analysis

All three readers performed image evaluation on a regular PACS workstation (Centricity, version 7.0; GE Healthcare, Solingen, Germany). DECT image analysis and statistical analysis were performed in accordance with the methods of a recently published study that assessed this algorithm for lumbar discs [27]. To establish a reference standard, two experienced and independent board-certified radiologists (I.Y. and T.V.), specialized in musculoskeletal imaging and with 7 and 36 years of expertise, respectively, reviewed the MRI images. In cases of discord, an additional board-certified radiologist (J.-E.S.) with 10 years of experience evaluated the MRI scan, and the prevailing consensus was documented for such cases.

Subjective assessments were conducted based on 5-point Likert scales to gauge diagnostic confidence (1: non-diagnostic, 2: low, 3: moderate, 4: high, 5: excellent) and image quality (1: insufficient, 2: weak, 3: moderate, 4: good, 5: excellent). Interquartile range (IQR) was determined for subjective assessment. The workstation’s preset windows were freely adjustable for image analysis.

Levels of degeneration in all thoracic intervertebral discs were categorized using the Pfirrmann grading system and categorized as follows: Pfirrmann grade 1 or 2 = no/mild degeneration, Pfirrmann grade 3 or 4 = moderate degeneration, Pfirrmann grade 5 = severe degeneration [27]. Following the reference standard, three blinded radiologists, each with varying levels of expertise in musculoskeletal imaging (reader 1, L.G., radiology resident, 4 years of experience; reader 2, C.B., board-certified radiologist, 7 years of experience; reader 3, V.K., radiology resident, 4 years of experience), independently assessed exclusively the DECT-derived color-coded collagen maps (including all available sequences) to evaluate the degree of thoracic disc degeneration based on the Pfirrmann grading system. All readers were blinded to the patients’ clinical records and reports.

### Statistical analysis

SPSS Statistics for MAC, version 29.0.1.0 (IBM, Armonk, NY) and Stata (version 13, StataCorp, College Station, TX) were used for statistical analysis. Continuous variables were presented as mean ± standard deviation, while categorical variables were represented as percentages. The normality of the data distribution was assessed using the Kolmogorov–Smirnov test. For normally distributed data, an unpaired *t*-test was performed, whereas the Wilcoxon Signed-Ranked test was utilized for non-normally distributed data.

Sensitivity (recall), F1-score as well as accuracy of DECT collagen maps were determined, with MRI as the reference standard for all examinations; point values and 95% confidence intervals (CIs) were calculated.

Intraclass correlation coefficient (ICC) was used in a two-way mixed-effects model to measure interobserver agreement among all radiologists and was interpreted according to the study by Koo et al: ICC < 0.50: poor agreement, ICC 0.50–0.75: moderate agreement, ICC 0.75–0.90: good agreement, ICC > 0.9: excellent agreement [28].

Statistical significance was considered at a *p*-value lower than 0.05.

## Results

**Fig. 2:**
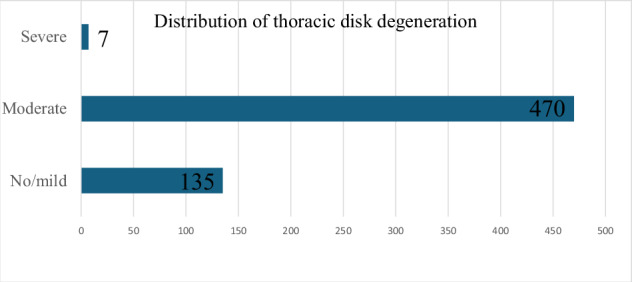
Degree of thoracic disc degeneration. Bar chart of the degree of thoracic disc degeneration across all evaluated thoracic discs. Degree of disc degeneration was categorized using the Pfirrmann grading system and categorized as follows: Pfirrmann grade 1 or 2 = no/mild degeneration, Pfirrmann grade 3 or 4 = moderate degeneration, Pfirrmann grade 5 = severe degeneration

In total, we examined 612 thoracic intervertebral discs in 51 patients aged 68 ± 16 years (mean ± standard deviation), 23 females and 28 males. MRI scans revealed 135 no/mildly degenerated discs (22.1%), 470 moderately degenerated discs (76.8%), and 7 severely degenerated discs (1.1%). The distribution of thoracic disc degeneration severity is illustrated in Fig. 2. Mean average time for reconstruction of collagen maps was 4 min (range, 3–5 min). Essential patient and clinical characteristics at baseline are outlined in Table 1.

**Table 1:** Patient characteristics

Parameters	Value
Number of patients	51
Male (*n*)/female (*n*)	28/23
Age at the date of MRI scan, years (mean ± standard deviation)	68 ± 16
Number of thoracic discs (*n*)	612
Degree of thoracic disc degeneration (*n*)	
No/mild (Pfirrmann 1 or 2)	135
Moderate (Pfirrmann 3 or 4)	470
Severe (Pfirrmann 5)	7

*MRI* Magnetic resonance imaging

### Subjective image quality per intervertebral disc

The subjective assessment of our reference standard (MRI) showed excellent image quality and diagnostic confidence in evaluating thoracic disc degeneration (image quality, median 5, IQR 5–5; diagnostic confidence, median 5, IQR 5–5). Qualitative evaluations of DECT-based color-coded collagen maps demonstrated high diagnostic confidence (median 4, IQR 3–4) as well as good image quality (median 4, IQR 4–4). The interreader reliability for the subjective assessment of DECT collagen maps was good for both diagnostic confidence (ICC = 0.82) and image quality (ICC = 0.80). Values of subjective analysis of thoracic disc degeneration are summarized in Table 2.

**Table 2:** Subjective analysis

Imaging modality	Diagnostic confidence, median (IQR)	Image quality, median (IQR)
Magnetic resonance imaging	5 (5–5)	5 (5–5)
Collagen maps		
All readers*	4 (3–4)	4 (4–4)
Reader 1	4 (4–4)	4 (4–4)
Reader 2	4 (3–4)	4 (4–4)
Reader 3	4 (3–4)	4 (4–4)

Values for subjective analysis of thoracic disc degeneration assessment. Five-point Likert scales were used for subjective evaluation to evaluate diagnostic confidence (1: non-diagnostic, 2: low, 3: moderate, 4: high, 5: excellent) and image quality (1: insufficient, 2: weak, 3: moderate, 4: good, 5: excellent)

* Intraclass correlation coefficient for all readers was 0.82 for diagnostic confidence and 0.80 for image quality

### Overall diagnostic accuracy per intervertebral disc

Overall analysis of DECT collagen maps revealed a recall (sensitivity) of 331/405 (81.7%) to detect no/mild thoracic disc degeneration, 1,134/1,410 (80.4%) to detect moderate disc degeneration, and 18/21 (85.7%) to detect severe disc degeneration. Overall F1-score was 71.0% for the diagnosis of no/mild disc degeneration detection, 86.5% for the diagnosis of moderate disc degeneration, and 30.5% for the diagnosis of severe disc degeneration. Overall accuracy for evaluating thoracic disc degeneration using collagen maps was 1,483/1,836 (80.8%). Interrater agreement for assessing thoracic disc degeneration based on Pfirrmann criteria using collagen maps was good (ICC = 0.89, 95% CI 0.87–0.90).

When comparing the findings of each reader, the most experienced reader (reader 2) yielded the highest accuracy value with an accuracy of 503/612 (82.2%), whereas the less experienced readers (rater 1 and 3) achieved lower accuracy values: reader 1, 490/612 (80.1%), reader 3, 490/612 (80.1%). Specifically, rater 1 yielded a sensitivity of 105/135 (77.8%) to discriminate no/mild disc degeneration, a sensitivity of 379/470 (80.6%) to discriminate moderate disc degeneration, and a sensitivity of 6/7 (85.7%) to discriminate severe disc degeneration. The most experienced reader (reader 2) achieved sensitivity values of 115/135 (85.2%) for no/mild disc degeneration, 382/470 (81.3%) for moderate disc degeneration, and 6/7 (85.7%) for severe disc degeneration. Reader 3 achieved a sensitivity of 111/135 (82.2%) for no/mild disc degeneration, 373/470 (79.4%) for moderate disc degeneration, and 6/7 (85.7%) for severe disc degeneration.

**Fig. 3:**
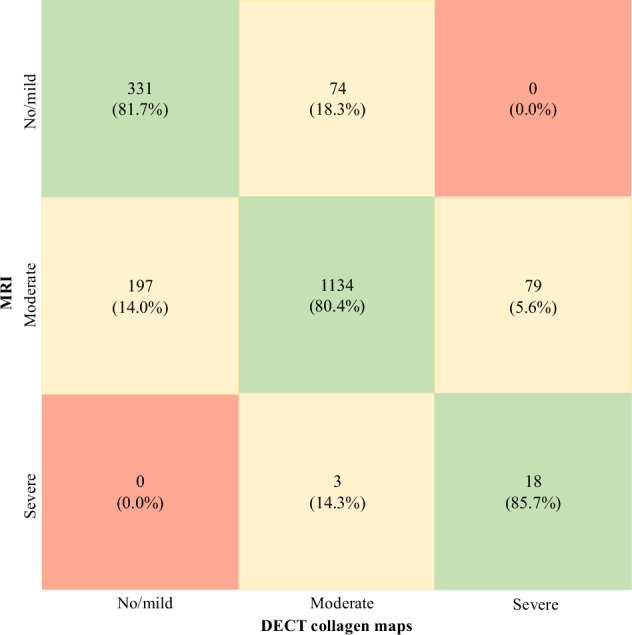
Confusion matrix for objective image analysis of DECT-based collagen maps. Confusion matrix with absolute numbers and percentages in parenthesis for objective image analysis to evaluate thoracic disc degeneration. Color serves as an indicator of the correctness of matches within each cell. DECT, Dual-energy computed tomography

**Fig. 4:**
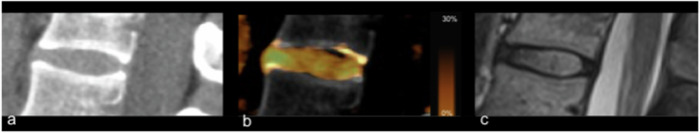
Sagittal reconstructions across a non/mildly degenerated thoracic disc between T12 and L1 in a 46-year-old male patient: **a** standard gray-scale computed tomography; **b** DECT collagen map; and **c** T2-weighted MRI. DECT, Dual-energy computed tomography; MRI, Magnetic resonance imaging

**Fig. 5:**
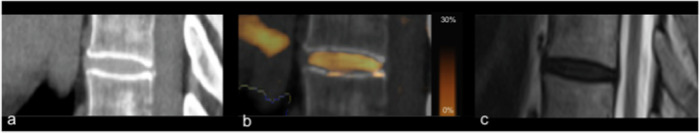
Sagittal reconstructions across a moderately degenerated thoracic disc between T9 and T10 in a 55-year-old female patient: **a** standard gray-scale computed tomography; **b** DECT collagen map; and **c** T2-weighted MRI. DECT, Dual-energy computed tomography; MRI, Magnetic resonance imaging

**Fig. 6:**
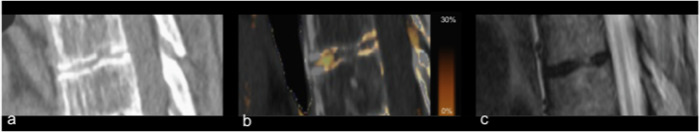
Sagittal reconstructions across a severely degenerated thoracic disc between T11 and T12 in a 75-year-old male patient: **a** standard gray-scale computed tomography; **b** DECT collagen map; and **c** T2-weighted MRI. DECT, Dual-energy computed tomography; MRI, Magnetic resonance imaging

**Fig. 7:**
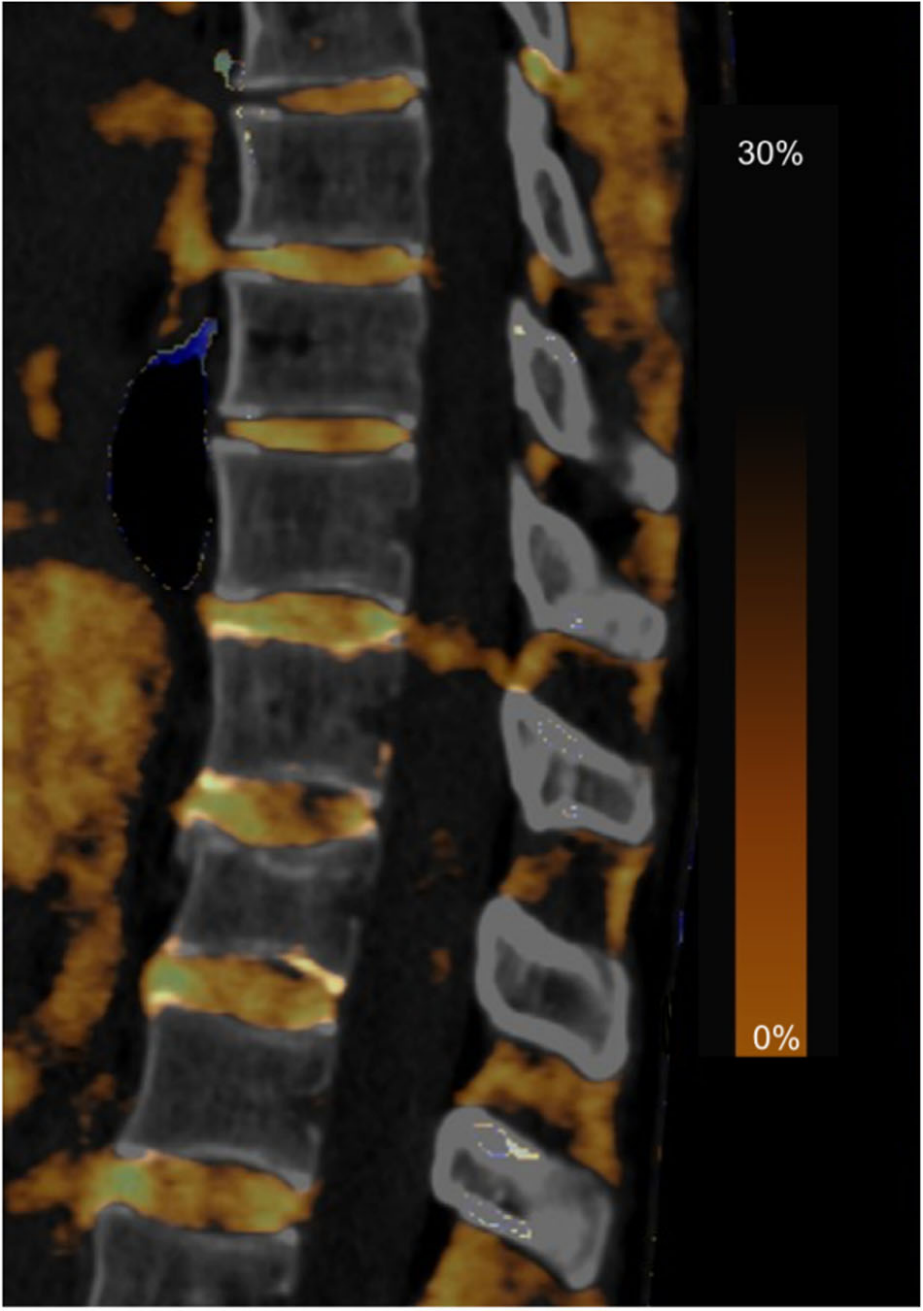
Sagittal DECT collagen map across the thoracolumbar section in a 52-year-old male patient with a compression fracture of T12, including vertebrae from T7 to L2 showing non/mildly and moderately degenerated thoracic discs. The uppermost disc (T7/T8) was considered moderately degenerated by all readers, whereas all other discs (from T8 to L2) were rated as non/mildly degenerated by all readers

Diagnostic accuracy values and confusion matrix for DECT-based collagen maps across all readers are displayed in Table 3 and Fig. 3. Figures 4–7 demonstrate DECT collagen maps across different degrees of disc degeneration in comparison with standard gray-scale CT and MRI.

**Table 3:** Diagnostic accuracy of collagen maps for thoracic disc degeneration assessment

Diagnostic accuracy	Recall (sensitivity)			F1-score			Accuracy
Thoracic disc degeneration	No/mild	Moderate	Severe	No/mild	Moderate	Severe	
Overall	81.7% (95% CI, 77.9–85.5%) 331/405	80.4% (95% CI, 78.3–82.5%) 1,134/1,410	85.7% (95% CI, 70.2–94.2%) 18/21	71.0%	86.5%	30.5%	80.8% 1,483/1,836
ICC	0.89 (95% CI, 0.87–0.90)						
Reader 1	77.8% (95% CI, 70.6–85.0%) 105/135	80.6% (95% CI, 77.0–84.2%) 379/470	85.7% (95% CI, 59.1–97.7%) 6/7	69.3%	86.1%	29.3%	80.1% 490/612
Reader 2	85.2% (95% CI, 79.2–89.2%) 115/135	81.3% (95% CI, 77.0–85.6%) 382/470	85.7% (95% CI, 59.1–97.7%) 6/7	73.7%	87.5%	30.8%	82.2% 503/612
Reader 3	82.2% (95% CI, 75.7–88.7%) 111/135	79.4% (95% CI, 76.0–82.8%) 373/470	85.7% (95% CI, 59.1–97.7%) 6/7	69.8%	86.0%	31.6%	80.1% 490/612

Values for objective analysis of thoracic disc degeneration assessment, including recall (sensitivity), F1-score and accuracy

*CI* Confidence interval, *ICC* Intraclass correlation coefficient

## Discussion

Our purpose was to investigate the diagnostic accuracy of DECT-derived color-coded collagen maps to assess thoracic disc degeneration. Our findings revealed high image quality and good diagnostic confidence for thoracic disc degeneration assessment using DECT collagen maps with a median score of 4 (IQR 4–4 and 3–4, respectively) on a 5-point Likert scale. Overall accuracy for evaluating thoracic disc degeneration with the Pfirrmann grading system was 80.8% compared to the reference standard MRI. The interrater agreement was good (ICC 0.89), emphasizing the method’s reliability. Our results thus promote the application of DECT for thoracic disc degeneration assessment, especially in patients with contraindications for MRI or in cases where MRI is not available. To the best of our knowledge, this is the first study to evaluate the application of DECT-derived collagen maps for thoracic disc degeneration assessment.

Over recent years, several studies have explored the application of DECT and its various postprocessing methods in the context of spinal pathologies. For instance, in a study using DECT-derived virtual non-calcium reconstructions for lumbar disc herniation assessment, the algorithm enabled a higher sensitivity and specificity (sensitivity 91% and specificity 92%) compared to standard gray-scale CT alone (sensitivity 80% and specificity 85%) [29]. Encouraging results were also observed in a comparable study from 2021 concerning thoracic disc herniation assessment, where sensitivity and specificity achieved 94 and 96%, respectively [30]. With regard to DECT-derived collagen maps, two studies investigated their diagnostic value for evaluating degenerative change of lumbar discs. Schömig et al revealed a sensitivity of 0.87 to detect lumbar disc pathology with DECT-derived collagen maps in 21 discs in 13 patients using MRI as reference standard and further demonstrated an improved interrater agreement compared to conventional CT (ICC 0.65 *versus* 0.32) [23]. Similarly, Abdellatif et al found an increased sensitivity (96.6% *versus* 87.2%) for diagnosing lumbar disc extrusions and sequestrations when implementing an additional collagen decomposition algorithm compared to standard CT alone in a group of 42 patients, again using MRI as a reference standard [24]. A study by Pumberger et al investigated the value of DECT for detecting disc injuries in the context of thoracolumbar vertebrae fractures in 295 discs in 67 patients over 50 years of age and demonstrated a high sensitivity of 0.85 as well as an improved interrater agreement (Fleiss’s *κ* 0.51 *versus* 0.41) compared to the reference standard MRI [25]. The high interrater agreement was also remarkable in our study (ICC 0.89), suggesting straightforward image analysis and potentially indicating an advantage of DECT over the more complex assessment of multiple MRI sequences, particularly when evaluated by less experienced readers. Our findings thus further complement the conclusions of comparable studies. Overall, these results suggest that DECT can provide added value for detecting various disc pathologies in different clinical settings, especially in cases where MRI is not applicable.

While our study offers valuable perspectives on DECT collagen mapping, it is important to address its limitations. First, the single-center design and retrospective nature pose the risk of introducing biases, thus further prospective and multi-center studies with larger and adjusted cohorts are needed to validate our findings. Second, our exclusion criteria, which include known or suspected malignancies and acute inflammatory processes, might unintentionally leave out certain relevant subgroups when studying thoracic disc degeneration. This could potentially limit the general applicability of our findings. Third, the level of experience in cross-sectional imaging among the readers differed within the two analyzed image techniques (DECT collagen maps *versus* MRI), which may have caused bias due to the varying levels of reader experience. Last, the technical method we employed relies on a dual-source DECT system, which means our findings may not be directly applicable to imaging departments without access to such equipment. For this purpose, a direct comparison between DECT collagen maps and standard gray-scale CT might have enhanced the quality of our study.

In conclusion, this is the first paper to suggest that DECT-derived color-coded collagen maps can present a valuable tool in assessing degenerative changes of thoracic discs. We demonstrated a high accuracy (80.8%) as well as a good interrater agreement for thoracic disc degeneration assessment using DECT-derived collagen maps. Although MRI remains the most comprehensive imaging modality in this respect, it may not be suitable for all patients. Especially for these cases, our results, in conjunction with supportive data from related studies, further promote the use of DECT-derived collagen maps for the assessment of thoracic disc pathologies. Additionally, DECT-derived collagen maps could provide valuable opportunistic evaluation of disc degeneration when CT scans are performed for different indications.

## Data Availability

The datasets used and/or analyzed during the current study are available from the corresponding author upon reasonable request.

## Abbreviations

CI: Confidence interval
CT: Computed tomography
DECT: Dual-energy computed tomography
ICC: Intraclass correlation coefficient
IQR: Interquartile range
MRI: Magnetic resonance imaging
